# Influence of Parent Stressors on Adolescent Obesity in African American Youth

**DOI:** 10.1155/2019/1316765

**Published:** 2019-12-01

**Authors:** Lauren Allport, MinKyoung Song, Cindy W. Leung, Kellye C. McGlumphy, Rebecca E. Hasson

**Affiliations:** ^1^University of Michigan, Childhood Disparities Research Laboratory, Ann Arbor, MI, USA; ^2^Oregon Health & Science University, School of Nursing, Portland, OR, USA; ^3^University of Michigan, School of Nursing, Ann Arbor, MI, USA; ^4^University of Michigan, School of Public Health, Ann Arbor, MI, USA; ^5^University of Michigan, School of Kinesiology, Ann Arbor, MI, USA

## Abstract

**Objective:**

The purpose of this study was to examine the relationships between individual parent stressors (financial, legal, career, relationships, home safety, community safety, medical, housing, authority, and prejudice) and adolescent obesity in African American adolescents.

**Methods:**

Data were from a cross section convenience sample of 273 African American parent-child dyads (ages 11–19) from Washtenaw County, Michigan. A subset of 122 dyads who completed parent and child questionnaires were included in this analysis. Parent stressors were assessed using the Crisis in Family Systems Revised (CRISYS-R) questionnaire. Height, weight, and waist circumference were measured by trained staff; height and weight were converted to BMI. Multivariate linear regression models were used to examine the relationships between individual parent stressors and adolescent BMI and waist circumference.

**Results:**

Parental exposure to stressors related to safety in the community was positively associated with adolescent BMI (*β* = 1.20(0.47), *p*=0.01) and waist circumference (*β* = 2.86(1.18), *p*=0.02). Parental appraisal of stressors related to safety in the community as “difficult to get through” was positively associated with adolescent BMI (*β* **=** 0.39(0.14), *p*=0.006) and waist circumference (*β* = 1.00(0.35), *p*=0.005). These relationships remained significant when adjusting for behavioral and psychosocial covariates. There were no significant relationships observed between other parent stressors and adolescent BMI or waist circumference.

**Conclusion:**

These findings suggest parents' exposure and appraisal of stressors related to community safety are associated with increased adolescent obesity in African American youth. Longitudinal, larger-scale studies are needed to better understand the mechanisms by which community safety may increase obesity risk in this ethnic minority pediatric population. This trail is registered with NCT02938663.

## 1. Introduction

Childhood obesity is a major public health concern, particularly among African American youth. Data from the National Center for Health Statistics estimated that 22% of African American youth were classified as obese compared to 14% of non-Latino White youth [1]. Psychological stress—an individual's appraisal of a situation as threatening when the demands of the situation are greater than the resources available—has been identified as a contributing factor to this obesity disparity [2]. In the *Health and Behaviour in Teenagers Study*, Van Jaarsveld and colleagues noted that higher levels of psychological stress over 5 years of follow-up was associated with higher waist circumference and body mass index (BMI) in a multiethnic sample of 4,065 adolescents (ages 11–16) [3]. In the *National, Heart, Lung, and Blood Institute Growth and Health Study,* Tomiyama and colleagues observed that higher levels of psychological stress over 10 years of follow-up predicted larger increases in BMI in 2,379 African American and White adolescent girls (ages 10–19) [4]. Nelson and colleagues identified specific stressors—social and environmental circumstances that produce psychological stress—and observed cross-sectional associations between low household education, exposure to racial discrimination, and BMI in 198 African American adolescents (ages 11–19) [5]. Taken together, these findings suggest that both stress appraisals (i.e., psychological stress) and stress exposure (i.e., stressors) can directly influence obesity risk in African American youth.

While research has shown a consistent relationship between parent stress and child weight status, few have explored the independent roles of specific stressors on adolescent obesity in African American youth. In the *Children's Health Study*, Shankardass and colleagues examined the impact of parental psychological stress on BMI in 4,078 children (ages 5–10 years) over four years of follow-up [6]. Study participants were majority Latino and non-Latino White. Parental stress at baseline was associated with a small but significant increase in predicted adolescent BMI at age 10. Increases in parental stress were also associated with an increased trajectory of weight gain in youth over follow-up. Using data from the *Southeastern Pennsylvania Household Health Survey,* Parks and colleagues determined that the number of stressors experienced by parents was related to child obesity in a sample of 2,119 parents/caregivers and their children (ages 3–17) [7]. This sample included Latino, African American, and non-Latino White youth; however, most participants were Latino. Koch and colleagues examined the role of parent stress and child obesity in a sample of 7443 Swedish families from the *All Babies in Southeast Sweden project* [8]. This study demonstrated that children from families that reported stress from multiple domains (e.g., relationships and health issues) had higher adjusted odds ratios for obesity, cross-sectionally (OR, 2.1; *p* < 0.01) and longitudinally (OR, 2.6; *p* < 0.01) [8].

Different types of parent stressors are likely to influence adolescent obesity in a variety of ways. For example, financial stressors—changes in income and inability to obtain household goods—could contribute to the types of food parents are able to buy, leading to low-cost, but high-calorie foods in the home. In the same way, financial stressors may make purchasing physical activity equipment or paying fees associated with sports team participation a challenge [9]. Stress related to safety issues in the community may reduce opportunities for adolescents to be physically active due to parent worry [10]. Stress related to medical issues pertaining to others where the parent becomes the primary caregiver of a family member or friend may also lead to prolonged periods of stress and contribute to the parent choosing less healthy food options [11, 12]. Stress related to prejudice—being treated unfairly due to one's social identity—or conflicts with authority may lead to both emotional distress in the parent and child and a decreased focus on parenting [13]. As such, identifying parent stressors can help disentangle the specific pathways leading to higher BMI and waist circumference in adolescents and provide important information for developing family-based interventions that reduce obesity in African American adolescents.

As few studies have examined parent stressors comprehensively, the aim of this study was to examine parent stressors from eleven different content areas including financial, legal, career, relationships, safety in the home, safety in the community, medical issues pertaining to self, medical issues pertaining to others (i.e., children, family, or friends), home issues (i.e., moving homes, changes in home occupants, and housing quality), authority (i.e., conflict with authority figures including health professionals, teachers, and supervisors), prejudice, and their influence on adolescent BMI and waist circumference among African American adolescents. We hypothesized that both exposures to and appraisals of each type of stressor experienced by the parent would predict higher BMI and waist circumference in their children.

## 2. Methods

### 2.1. Sample Population, Recruitment, and Procedure

Adolescents were recruited from neighboring communities in southeast Michigan to participate in the Health and Culture Project, a two-component study investigating social, psychological, and cultural predictors of obesity [5, 14–16]. The first component involved adolescents and their parents completing questionnaires assessing psychological stress, socioeconomic status, culture, and other environmental factors and included objective measures of physical activity via accelerometry, dietary assessments, and anthropometric measurements. The second component provided information to adolescents about their own health behaviors, which was followed by a researcher-led discussion with adolescent participants about ways in which they could integrate healthy eating and physical activity into their lives. Individuals were excluded from participating in this study if they had participated in a weight loss or exercise program within the previous 6 months, were taking any medications known to affect body weight, or were diagnosed with any syndrome or disorder that would affect body weight or any other major illness. Adolescents were also excluded if they were diagnosed with clinical depression or any other mental health disorder that may influence mood, emotions, or stress perception.

Two hundred seventy-three adolescent participants, ages 11–18 years, were recruited from middle schools (*n* = 85), high schools (*n* = 31), after-school programs (*n* = 46), community centers (*n* = 30), and the general community in Washtenaw County, Michigan (*n* = 81), via flyers and word of mouth. Data collection occurred at the University of Michigan Childhood Disparities Research Laboratory or off-site at a school, after-school program, or community center. In total, 122 African American adolescents (76 girls, 46 boys) who completed questionnaires assessing food intake and had at least 4 days of valid accelerometer data were included in this analysis. Participants were excluded for the following reasons: 20 child participants did not self-identify as African American or Black, 19 child participants were outliers for caloric intake (i.e., <500 and >3500 kcal/day), and 109 caregivers did not complete the stress questionnaire [17]. The parents of the 122 adolescent participants completed a parent questionnaire, so a total of 122 dyads (parent and child) were included in this analysis. When adolescent participants who were included in this analysis (*n* = 122) were compared with those who were excluded (*n* = 151), both groups were similar in BMI, height, weight, and waist circumference (data not shown, *p*'s > 0.05). Included participants, however, were older (15.0 ± 0.18 vs. 14.0 ± 0.15 years old; *p* < 0.01) compared with excluded participants. Written assent (for adolescents under age 17) and written consent from older adolescents and parents were obtained before participating. This study was reviewed and approved by the University of Michigan Institutional Review Board.

### 2.2. Dependent Variables

#### 2.2.1. Body Mass Index

BMI was calculated using guidelines from the Centers for Disease Control and Prevention [18]. Body weight (kg) and height (cm) were measured to the nearest 0.1 kg and nearest 0.1 cm, respectively, using an electronic scale (Doran Scales, Inc., Batavia, IL) and ShorrBoard® (Weigh and Measure, LLC, Olney, MD). Both body weight and height were measured twice, and the average of the two measurements was used to calculate BMI. All measurements were completed using standardized procedures [19].

#### 2.2.2. Waist Circumference

Waist circumference was measured using guidelines from the Centers for Disease Control and Prevention (CDC) [20]. Waist circumference (cm) was measured above the iliac crest to the nearest 0.1 cm using a Gulick tape measure [20]. Measurements were taken twice, and if measurements were not within 5 mm of each other, they were retaken. Once two measurements were obtained, the average was calculated and recorded.

### 2.3. Independent Variables

Parent stressors were measured using the Crisis in Family Systems Revised (CRISYS-R) questionnaire which has been validated for use in a multiethnic sample including African American parents [21]. This 63-item survey asks parents about stressors they have experienced from eleven different categories including financial, legal, career, relationships, safety in the home, safety in the community, medical issues pertaining to self, medical issues pertaining to others, home issues, authority, and prejudice. Definitions for each parent stressor are included in Table 1. For each category, the parent selected whether the stressor occurred in the last 6 months by circling either “yes” or “no”. Parents then rated the difficulty of the stressor experience on a 5-point Likert scale ranging from 1 “was not difficult to get through” to 5 “was extremely difficult to get through.” For example, one item on the survey read, “Did anything happen in your neighborhood that made you feel unsafe? If yes, was it difficult to get through?” Exposure scores were calculated by summing the yeses for each stressor category. Appraisal scores were calculated by summing the difficulty ratings for each stressor category. For example, if a parent answered “yes” to 3 out of 8 questions in the community safety category and rated each of those questions a difficulty of “5” to get through, their exposure score would be 3 and their appraisal score would be 15. For parents who answered “no,” their exposure score would be 0 and their appraisal score would be 0.

### 2.4. Covariates

Adolescent's sex and age were assessed via child self-report. Moderate-to-vigorous physical activity (MVPA) and sedentary activity was assessed over a 7-day period for each participant via accelerometry (GT3XActiLife, ActiGraph, Pensacola, FL). Participants were asked to wear the device snugly on their right hip using an adjustable belt. At home, they were advised to remove the accelerometers during bathing, contact sports, water-based activities, and sleeping. Participants were also asked to complete a nonwear and sleep diary. The frequency at which the devices collected raw data was 30 Hz. The raw data obtained from the accelerometer were retrieved and integrated into 10-second epochs using ActiLife software, version 6.11.8. At least 4 days of valid (>600 min/day) accelerometer data were required for MVPA and sedentary activity to be recorded. Puyau cut-points, most commonly used for adolescent populations, were used to derive moderate-to-vigorous activity intensity [22]. These cut-points were selected because Freedson cut-points often overestimate exercise intensity by misclassifying light physical activity as MVPA [23]. Total daily caloric intake was collected using the 2012 Youth/Adolescent Food Frequency Questionnaire (YAQ) developed by Harvard University [24]. The FFQ assesses nutrient and energy intake by asking questions regarding the frequency of how much the participant consumed specific foods and drinks throughout the preceding year. The Harvard T. H. Chan School of Public Health analyzed the FFQ data using the Nutrition Data System for Research (NDSR) Windows-based dietary analysis program [25]. This questionnaire was designed, validated, and calibrated for use in adolescents [26]. Parent's employment status was measured via self-report. Parents who answered as unemployed or retired were coded as 0, and parents who were currently employed were coded as 1. Given the confounding effects of adolescent sex, age, physical activity, sedentary time, parent employment status, and dietary intake on the relationships between parent stress and adolescent obesity, these variables were included in the regression models as covariates [27, 28].

### 2.5. Statistical Analyses

Before analysis, data were evaluated for normality, and natural log transformations were made to the following variables: dietary intake, sedentary time, and MVPA. Spearman's correlations were used to explore the associations between the parent stressor variables (financial, legal, career, relationships, home safety, community safety, medical, housing, authority, and prejudice). Multiple imputation was used to account for missing data in both adolescents and parents. Most study variables had <10% missing data. The highest fraction of missing data was MVPA (29.2%). Participants with imputed data did not significantly differ by age, BMI, waist circumference, height, or weight from participants without imputed data (*p* > 0.05). To examine the relationship between parent stressors and adolescent BMI, two models were run for each stressor. The first model included the exposure score as the dependent variable, and the second model included the appraisal score as the dependent variable. Both age-adjusted and multivariate linear regression models were used to examine the relationship between parent stressors and BMI with adolescent sex, age, dietary intake, sedentary time, MVPA, and parent employment status included as covariates. The same analyses were then run to examine the relationship between parent stressors and waist circumference. The significance level for all analyses was *α* < 0.05. Analyses were conducted using Stata Special Edition 14.0.

## 3. Results

Participant characteristics are shown in Table 2. The average age of adolescent participants was 15 years, and average BMI was 25.6 kg/m^2^ with 13% of adolescent participants classified as overweight or obese based on BMI percentile. This is below the national average of 22% for African American youth aged 6–17 [29]. On average, adolescent participants engaged in 13 minutes of MVPA per day which is lower than the national average for African American adolescents of 20 minutes per day [30]. Adolescent participants consumed an average of 1800 calories a day which is less than the national average of 2100 calories a day [31]. Parents in the study had higher educational attainment as compared to national averages (61% had at least a bachelor's degree compared to 18% of African Americans nationally) [32]. In addition, compared to national averages, parents were more likely to be married (42.5% vs. national average 29.2%), more likely to be divorced (23% vs. national average 11.8%), and less likely to never have been married (29% vs. national average 50%) [33]. Overall, adolescent participants in this study had slightly lower rates of obesity, MVPA, and caloric intake, and their parents were more likely to have at least a college degree and be married or divorced and were less likely to never have been married. Thus, the generalizability of this study may be limited to African American adolescents residing in southeast Michigan.

Table 3 shows the number of parents who were exposed to each stressor, the average number of stressors experienced per category, and average appraisal scores for each stressor category. Parents reported an average of 6.5 stressors in the past six months. The most frequent stressors reported in rank order were financial, career, safety in the community, relationships, home issues, medical issues (others), prejudice, medical issues (self), legal, authority, and safety in the home.

Table 4 shows Spearman's correlations for stressor exposures. The strongest correlation was seen between prejudice and authority (*r* = 0.48, *p* < 0.05). The weakest correlation was seen between medical issues (self) and legal issues (*r* = 0.004, *p* > 0.05). Most correlations between parental stressor exposures were weak (*r* = 0.05–0.40).

Table 5 shows correlations for stressor appraisals. Appraisal of prejudice stressors was moderately correlated with appraisal of authority stressors (*r* = 0.54, *p* < 0.05). The weakest correlation was seen between home safety and authority (*r* = −0.005, *p* > 0.05). Most correlations between parental stressor appraisals were weak (*r* = 0.05–0.40).

Table 6 shows the regression models for parent stressor exposure and youth BMI and waist circumference. There was a trend towards significance in the age-adjusted model for exposure to stressors related to safety in the community and adolescent BMI with greater exposure to this stressor associated with increased adolescent BMI (*β* = 0.90(0.46), *p*=0.05). When sex, dietary intake, sedentary time, MVPA, and parent employment were accounted for, the association between stressors related to safety in the community and adolescent BMI became significant (*β* = 1.20(0.47), *p*=0.01). Exposure to stressors related to safety in the community was positively associated with adolescent waist circumference for both the age-adjusted model (*β* = 2.55(1.19), *p*=0.03) and the multivariate-adjusted model (*β* = 2.86(1.18), *p*=0.02).

Table 7 shows the regression models for parent stressor appraisals and youth BMI and waist circumference. Appraisal of stressors related to safety in the community was positively associated with youth BMI in both the age-adjusted model (*β* = 0.31(0.13), *p*=0.02) and multivariate-adjusted model (*β* = 0.39(0.14), *p*=0.006). Appraisal of stressors related to safety in the community was positively associated with adolescent waist circumference in the age-adjusted model (*β* = 0.94(0.34), *p*=0.006) and the multivariate-adjusted model (1.00(0.35), *p*=0.002). Appraisal of stressors related to parent medical issues was positively associated with adolescent waist circumference in the age-adjusted model (*β* = 1.43(0.70), *p*=0.045); however, this relationship was no longer significant after adjusting for sex, dietary intake, sedentary activity, MVPA, and parent employment status (*β* = 1.15 ± 0.70, *p*=0.10).

## 4. Discussion

This study explored different parent stressors and their relation to adolescent obesity in a sample of African American parent-child dyads. While previous studies assessed BMI as an indicator of adolescent obesity, this study examined both adolescent's BMI and waist circumference as previous data suggest stress plays a greater role in central adiposity (measured via waist circumference) than BMI [34]. Consistent with previous data, our findings demonstrate that parent stress related to safety in the community was positively associated with adolescent BMI and waist circumference. These relationships remained significant when considering the effects of adolescent sex, age, dietary intake, sedentary time, MVPA, and parent employment status. Parent appraisals of stressors related to their own medical issues were also positively associated with waist circumference, but this relationship did not remain significant with the addition of covariates. Contrary to our hypotheses, there were no significant relationships observed between the other nine parent stressors and adolescent BMI and waist circumference. Taken together, these results demonstrate the unique contribution of parent stress on adolescent obesity in African American youth.

Previous research supports our findings that parent stress related to safety in the community are associated with increased adolescent obesity. Lumeng et al. reported that parental perceptions of their neighborhood as unsafe was associated with a greater risk of being overweight by age 7 in a multiethnic sample of 768 children from the *National Institute of Child Health and Human Development Study of Early Child Care and Youth Development* [35]. Previous research conducted with ethnic minority families has also determined that mothers who perceive their neighborhoods as unsafe will limit their child's physical activity outdoors [36, 37]. Cecil-Karb and Grogan-Kaylor observed, in a multiethnic sample of 5,886 children between the ages of 5 and 20, a positive association between perceived neighborhood safety and adolescent BMI which was mediated by television viewing [38]. Cecil-Karb and Grogan-Kaylor concluded that parents who perceive their neighborhood as unsafe were more likely to promote indoor activities such as watching television rather than outdoor activities [38]. Yet, in the present study, the relationship between parent stress related to safety in the community and child BMI and waist circumference remained significant when accounting for both child MVPA and sedentary time. This indicates that parent stress related to neighborhood safety may influence adolescent weight through other mechanisms outside of parent promotion of sedentary activity. Additional research is warranted to determine how parent stress related to neighborhood safety influences adolescent BMI and waist circumference.

In the age-adjusted models, parent stressors related to personal medical issues were positively associated with adolescent waist circumference. These findings are consistent with Isasi and colleagues who included parent health in overall measures of stress and found a positive association between parent stress and adolescent obesity [39]. Garasky and colleagues explored the influence of parents' physical and mental health problems on adolescent obesity and reported a positive association between the two in a sample of multiethnic adolescents ages 12–17 [40]. Findings in the present study provide preliminary evidence that the relationship between parent stressors related to medical issues and waist circumference may in part be explained by adolescent health behaviors including MVPA, sedentary time, and dietary intake. In our multivariate regression models, stressors related to parent medical issues were no longer significantly associated with adolescent waist circumference after accounting for these health behaviors. If the stress related to the parent's health status is directly or indirectly affecting adolescent health behaviors, interventionists should consider developing health education programming during this critical time to help both parents and adolescents cope with the stress of a new diagnosis or medical complication. Additional research is warranted.

There were no significant relationships between adolescent BMI or waist circumference with the remaining stressors. It is important to note that our sample had a higher level of educational attainment compared to African Americans nationally (61% had at least a bachelor's degree vs 18%) which may have served as a protective factor in this cohort [5, 32]. Education is the most stable indicator of socioeconomic status, and having more education can lead to increased health literacy which in turn enables parents to make more informed health decisions for their family [9]. Household education is also closely linked with employment opportunities and family income [9]. Employment opportunities provide greater access to medical care through work-based insurance, and family income provides parents with the ability to purchase health-related goods [9]. Overall, these resources associated with household educational attainment enable parents and caregivers to create healthy home environments and model healthy behaviors for their children. As such, the high level of household education in this cohort of African American parents may have reduced our ability to observe significant associations between parent stressors related to legal, career, relationships, housing, authority, and prejudice, and their child's weight status. Future research in a lower education cohort may be needed to better understand these relationships.

The strengths of this study include the focus on African American adolescents (a group at increased risk for childhood obesity), controls in the analyses for appropriate variables, objective measures of adolescent MVPA and sedentary time using accelerometers, the use of multiple measures of adolescent obesity (BMI and waist circumference), and the inclusion of a wide array of parent stressors using a validated measure. Limitations included a small convenient sample and a cross-sectional design that relied on self-reported data and the use of a food frequency questionnaire (FFQ) to measure adolescent caloric intake. High fractions of missing data were noted for adolescent physical activity; however, multiple imputation was used to account for these missing data. Additionally, the study used a wide range of ages (11–18) and had a high proportion of female participants (62%). Finally, the generalizability of this study may be limited to African American youth residing in Southeast Michigan given the higher level of household education reported in this group. Despite these limitations, we observed a strong and consistent relationship between parent stress related to safety in the community and adolescent waist circumference.

The present study suggests that parent stressors related to community safety can play an important role in shaping adolescent weight status among African American youth. Additionally, addressing the broader financial and safety concerns present in African American neighborhoods may also be useful in overcoming the potential risks imposed by an unsafe neighborhood on adolescent obesity. Indeed, findings from the *Moving to Opportunity* study [41] demonstrated that where a family lives plays an important role in shaping adolescent health. Families that moved from high-poverty/higher-crime neighborhoods to low-poverty/lower-crime neighborhoods were less likely to be obese after 4–7 years as compared to a control group. Interestingly, our study indicates that even in families with high educational attainment, the safety of one's neighborhood can have a lasting impact on obesity risk for African American adolescents. Public health planners should consider multilevel obesity prevention policies and interventions that address community safety in African American communities in their efforts to reduce obesity disparities in this pediatric population.

## Figures and Tables

**Table 1:** Definitions of stressors.

Stressors	Definition
Financial	Changes in income and inability to obtain resources such as food, clothing, housing, and transportation.
Legal	Interactions with the legal system including experiencing the arrest of a family member, or a family member going to jail.
Career	Getting laid off, changing jobs, or returning to school.
Relationships	Experiencing divorce, a break up, or death of a friend or family member.
Safety in the home	Feeling emotionally or physically unsafe in the home or experiencing a crime in the home.
Safety in the community	Hearing, witnessing, or experiencing crime in one's neighborhood or otherwise feeling unsafe.
Medical issues (self)	Personally experiencing a chronic illness, going to the hospital, or becoming pregnant.
Medical issues (others)	Having a child or family member become ill or be admitted to the hospital.
Home issues	Experiencing a loss of housing, change in home occupants, or issues with housing quality.
Authority	Conflict with authority figures including health professionals, teachers, and supervisors.
Prejudice	Being treated unfairly due to one's age, socioeconomic status, gender, or race.

**Table 2:** Participant characteristics.

	*n*	Mean ± SE	Percentage
Child characteristics			
Age (years)	122	14.92 ± 0.18	—
Weight (kg)	122	68.9 ± 1.67	—
Height (cm)	122	163.7 ± 0.82	—
BMI (kg/m^2^)	122	25.6 ± 0.57	—
BMI percentile	122	76.5 ± 2.17	—
Waist circumference (cm)	111	81.4 ± 1.58	—
MVPA (minutes/day)	85	12.60 ± 1.28	—
Dietary intake (kcals/day)	98	1791.89 ± 67.37	—
Sex	122	—	100
Female	76	—	62
Male	46	—	38

Parent characteristics			
Max parent education	119	—	100
Some high school	5	—	4
High school graduate/GED	14	—	12
Some college or vocational school	27	—	23
College graduate	39	—	33
Graduate or professional training	34	—	28
Relationship to child	120	—	100
Mother	102	—	85
Father	11	—	9
Other	7	—	6
Marital status	120	—	100
Single (never married)	35	—	29
Married	51	—	42.5
Divorced	28	—	23
Separated	3	—	2.5
Widowed	1	—	1
Living together	2	—	2
Employment status	117	—	100
Employed	107	—	91
Not working	10	—	9
Household income	114	—	100
<$20,000/year	25	—	22
$20,000–40,000/year	28	—	25
$40,000–60,000/year	23	—	20
$60,000–80,000/year	16	—	14
$80,000–100,000/year	13	—	11
>$100,000/year	9	—	8

*Note.* Data are mean ± SE. SE, standard error; BMI, body mass index; GED, general education development; MVPA, moderate-to-vigorous physical activity.

**Table 3:** Overview of parent stressors.

Stressors	Number of exposures (*n*)	Average appraisal (mean ± SE)
Financial	207	5.03 ± 0.74
Legal	43	1.06 ± 0.21
Career	93	1.92 ± 0.29
Relationships	74	2.04 ± 0.31
Safety in the home	17	0.45 ± 0.16
Safety in the community	76	1.73 ± 0.40
Medical issues (self)	47	1.04 ± 0.20
Medical issues (others)	63	1.71 ± 0.27
Home issues	67	1.66 ± 0.30
Authority	39	1.2 ± 0.24
Prejudice	61	1.57 ± 0.29

*Note.* Data are mean ± SE. SE, standard error.

**Table 4:** Correlations between exposure to stressors.

Correlations	1	2	3	4	5	6	7	8	9	10	11
(1) Financial	1 (118)										
(2) Legal	0.27^*∗*^ (118)	1 (121)									
(3) Career	0.29^*∗*^ (117)	0.29^*∗*^ (120)	1 (120)								
(4) Relationships	0.33^*∗*^ (117)	0.44^*∗*^ (120)	0.20^*∗*^ (120)	1 (120)							
(5) Home Safety	0.49^*∗*^ (117)	0.35^*∗*^ (120)	0.36^*∗*^ (119)	0.55^*∗*^ (119)	1 (120)						
(6) Com Safety	0.54^*∗*^ (117)	0.31^*∗*^ (116)	0.29^*∗*^ (115)	0.29^*∗*^ (115)	0.56^*∗*^ (115)	1.0 (116)					
(7) Med Self	0.38^*∗*^ (112)	0.23^*∗*^ (114)	0.17 (113)	0.31^*∗*^ (113)	0.20^*∗*^ (113)	0.37^*∗*^ (114)	1.0 (114)				
(8) Med Other	−0.02 (113)	0.05 (115)	−0.003 (114)	0.15 (114)	0.16 (114)	−0.08 (115)	0.21^*∗*^ (113)	1.0 (115)			
(9) Home issues	0.23^*∗*^ (116)	−0.003 (119)	0.09 (118)	0.08 (118)	0.13 (118)	0.22^*∗*^ (114)	0.19^*∗*^ (113)	0.16 (113)	1.0 (120)		
(10) Authority	0.17 (116)	0.06 (119)	−0.05 (118)	0.10 (118)	−0.04 (118)	0.04 (114)	0.20^*∗*^ (113)	0.27^*∗*^ (113)	0.33^*∗*^ (120)	1.0 (120)	
(11) Prejudice	0.18 (115)	0.05 (118)	0.08 (117)	0.12 (117)	0.28^*∗*^ (117)	0.15 (113)	−0.03 (112)	0.32^*∗*^ (112)	0.42^*∗*^ (119)	0.45^*∗*^ (119)	1.0 (119)

*Note.* Correlation coefficients are shown followed by sample size (*N*). Asterisk denotes significance at *p* < 0.05. Com Safety, community safety. Med Self, medical issues pertaining to self. Med Other, medical issues pertaining to others.

**Table 5:** Correlations between appraisals of stressors.

Correlations	1	2	3	4	5	6	7	8	9	10	11
(1) Financial	1 (118)										
(2) Legal	0.28^*∗*^ (118)	1 (121)									
(3) Career	0.41^*∗*^ (117)	0.24^*∗*^ (120)	1 (120)								
(4) Relationships	0.39^*∗*^ (117)	0.43^*∗*^ (120)	0.21^*∗*^ (120)	1 (120)							
(5) Home Safety	0.40^*∗*^ (117)	0.41^*∗*^ (120)	0.40^*∗*^ (119)	0.55^*∗*^ (119)	1 (120)						
(6) Com Safety	0.51^*∗*^ (117)	0.28^*∗*^ (116)	0.36^*∗*^ (115)	0.25^*∗*^ (115)	0.53^*∗*^ (115)	1.0 (116)					
(7) Med Self	0.36^*∗*^ (112)	0.34^*∗*^ (114)	0.15 (113)	0.34^*∗*^ (113)	0.18 (113)	0.33^*∗*^ (114)	1.0 (114)				
(8) Med Other	−0.002 (113)	0.15 (115)	−0.10 (114)	0.23^*∗*^ (114)	0.19^*∗*^ (114)	−0.04 (115)	0.31^*∗*^ (113)	1.0 (115)			
(9) Home issues	0.28^*∗*^ (116)	0.16 (119)	0.08 (118)	0.19^*∗*^ (118)	0.26^*∗*^ (118)	0.37^*∗*^ (114)	0.17 (113)	0.18 (113)	1.0 (120)		
(10) Authority	0.17 (116)	0.09 (119)	0.06 (118)	0.08 (118)	−0.03 (118)	0.11 (114)	0.15 (113)	0.28^*∗*^ (113)	0.34^*∗*^ (120)	1.0 (120)	
(11) Prejudice	0.16 (115)	0.18^*∗*^ (118)	0.15 (117)	0.21^*∗*^ (117)	0.43 (117)	0.32^*∗*^ (113)	−0.001 (112)	0.43^*∗*^ (112)	0.47^*∗*^ (119)	0.50^*∗*^ (119)	1.0 (119)

*Note.* Correlation coefficients are shown followed by sample size (*N*). Asterisk denotes significance at *p* < 0.05. Com Safety, community safety. Med Self, medical issues pertaining to self. Med Other, medical issues pertaining to others.

**Table 6:** Association between parent exposure to stressors and child waist circumference (*N* = 111) and BMI (*N* = 122).

Exposure	BMI^a^			Waist circumference		
Stressors	Β(SE)^b^	*p*	*R*-square	Β(SE)	*p*	*R*-square
Financial						
Age-adjusted	0.25(0.24)	0.31	0.02	0.79(0.63)	0.21	0.02
Multivariate-adjusted^c^	0.40(0.24)	0.11	0.19	1.21(0.65)	0.07	0.23
Legal						
Age-adjusted	0.64(0.86)	0.46	0.01	1.65(2.21)	0.46	0.01
Multivariate-adjusted	0.67(0.88)	0.44	0.18	1.91(2.23)	0.39	0.20
Career						
Age-adjusted	0.04(0.59)	0.94	0.006	0.79(1.56)	0.61	0.007
Multivariate-adjusted	0.26(0.60)	0.66	0.17	1.15(1.57)	0.47	0.20
Relationships						
Age-adjusted	0.31(0.56)	0.58	0.009	0.34(1.44)	0.81	0.005
Multivariate-adjusted	0.27(0.58)	0.64	0.17	0.48(1.44)	0.74	0.20
Safety in the home						
Age-adjusted	1.55(1.09)	0.16	0.02	3.02(2.77)	0.28	0.02
Multivariate-adjusted	2.37(1.24)	0.06	0.21	4.17(3.17)	0.19	0.21
Safety in the community						
Age-adjusted	0.90(0.46)	0.05	0.04	2.55(1.19)	0.03^*∗*^	0.05
Multivariate-adjusted	1.20(0.47)	0.01^*∗*^	0.22	2.86(1.18)	0.02^*∗*^	0.24
Stressor-adjusted^d^	0.78(0.57)	0.18	0.20	2.45(1.47)	0.10	0.21
Medical issues (self)						
Age-adjusted	0.09(0.77)	0.90	0.007	2.33(2.02)	0.25	0.02
Multivariate-adjusted	0.11(0.75)	0.89	0.17	2.15(1.96)	0.28	0.21
Medical issues (others)						
Age-adjusted	0.14(0.77)	0.86	0.008	0.54(1.96)	0.78	0.007
Multivariate-adjusted	−0.48(0.76)	0.53	0.17	−0.59(1.97)	0.76	0.20
Home issues						
Age-adjusted	−1.19(0.64)	0.07	0.03	−1.30(1.66)	0.43	0.01
Multivariate-adjusted	−0.81(0.67)	0.24	0.18	0.001(1.76)	0.99	0.19
Authority						
Age-adjusted	0.18(0.89)	0.84	0.007	−0.39(2.29)	0.87	0.005
Multivariate-adjusted	0.05(0.87)	0.95	0.17	−0.90(2.17)	0.68	0.19
Prejudice						
Age-adjusted	0.81(0.62)	0.20	0.02	−0.23(1.63)	0.89	0.005
Multivariate-adjusted	0.97(0.64)	0.13	0.19	−0.07(1.69)	0.97	0.19

*Note.* Standard beta coefficients with standard error are shown. ^*∗*^*p* < 0.05. ^a^Body mass index. ^b^Standard error. ^c^The multivariate-adjusted model included child age, sex, moderate-to-vigorous physical activity, sedentary time, caloric intake, and parent employment status. ^d^The stressor-adjusted model included financial, home safety, age, sex, moderate-to-vigorous physical activity, sedentary time, caloric intake, and parent employment status.

**Table 7:** Association between parent appraisal of stressors and child waist circumference (*N* = 111) and BMI (*N* = 122).

Appraisal	BMI^a^			Waist circumference		
Stressors	Β(SE)^b^	*p*	*R*-square	Β(SE)	*p*	*R*-square
Financial						
Age-adjusted	0.08(0.07)	0.27	0.02	0.24(0.19)	0.22	0.02
Multivariate-adjusted^c^	0.12(0.07)	0.10	0.17	0.33(0.19)	0.08	0.22
Legal						
Age-adjusted	0.08(0.25)	0.74	0.007	0.07(0.63)	0.91	0.004
Multivariate-adjusted	0.10(0.25)	0.69	0.16	0.13(0.63)	0.84	0.19
Career						
Age-adjusted	0.20(0.18)	0.27	0.02	0.51(0.49)	0.30	0.01
Multivariate-adjusted	0.27(0.19)	0.14	0.17	0.56(0.48)	0.25	0.20
Relationships						
Age-adjusted	0.07(0.17)	0.67	0.008	0.02(0.44)	0.96	0.004
Multivariate-adjusted	0.09(0.18)	0.61	0.16	0.07(0.45)	0.87	0.19
Safety in the home						
Age-adjusted	0.39(0.33)	0.24	0.02	0.82(0.84)	0.33	0.01
Multivariate-adjusted	0.64(0.39)	0.10	0.18	1.04(1.00)	0.30	0.20
Safety in the community						
Age-adjusted	0.31(0.13)	0.02^*∗*^	0.05	0.94(0.34)	0.006^*∗*^	0.08
Multivariate-adjusted	0.39(0.14)	0.006^*∗*^	0.22	1.00(0.35)	0.005^*∗*^	0.26
Stressor-adjusted^d^	0.28(0.16)	0.08	0.19	1.01(0.41)	0.02^*∗*^	0.23
Medical issues (self)						
Age-adjusted	0.40(0.28)	0.15	0.03	1.43(0.70)	0.045^*∗*^	0.05
Multivariate-adjusted	0.24(0.27)	0.38	0.16	1.07(0.69)	0.13	0.21
Medical issues (others)						
Age-adjusted	0.24(0.20)	0.23	0.02	0.56(0.51)	0.28	0.02
Multivariate-adjusted	0.06(0.21)	0.79	0.16	0.29(0.55)	0.60	0.19
Home issues						
Age-adjusted	−0.19(0.17)	0.26	0.02	−0.24(0.44)	0.60	0.007
Multivariate-adjusted	−0.11(0.19)	0.58	0.16	0.17(0.50)	0.74	0.19
Authority						
Age-adjusted	0.14(0.22)	0.53	0.01	0.18(0.57)	0.75	0.005
Multivariate-adjusted	0.10(0.22)	0.65	0.16	0.02(0.55)	0.97	0.19
Prejudice						
Age-adjusted	0.34(0.18)	0.07	0.03	0.45(0.48)	0.36	0.01
Multivariate-adjusted	0.36(0.19)	0.07	0.19	0.45(0.52)	0.39	0.20

*Note.* Standard beta coefficients with standard error are shown. ^*∗*^*p* < 0.05. ^a^Body mass index. ^b^Standard error. ^c^The multivariate-adjusted model included child age, sex, moderate-to-vigorous physical time, sedentary time, caloric intake, and parent employment status. ^d^The stressor-adjusted model included financial, home safety, age, sex, moderate-to-vigorous physical activity, sedentary time, caloric intake, and parent employment status.

## Data Availability

The participant data used to support the findings of this study are available from the corresponding author upon request.
